# Educational attainment and trajectories at key stages of schooling for children with amblyopia compared to those without eye conditions: Findings from the Millennium Cohort Study

**DOI:** 10.1371/journal.pone.0283786

**Published:** 2023-03-30

**Authors:** Lisanne Andra Horvat-Gitsels, Mario Cortina-Borja, Jugnoo Sangeeta Rahi

**Affiliations:** 1 Population, Policy and Practice Research and Teaching Department, Great Ormond Street Institute of Child Health, University College London, London, United Kingdom; 2 Ulverscroft Vision Research Group, Great Ormond Street Institute of Child Health, University College London, London, United Kingdom; 3 Great Ormond Street Hospital for Children NHS Foundation Trust, London, United Kingdom; 4 Institute of Ophthalmology, University College London and NIHR Moorfields Biomedical Research Centre London, London, United Kingdom; Al-Ahliyya Amman University, JORDAN

## Abstract

**Background:**

Amblyopia is a neurodevelopmental condition resulting in reduced vision for which whole population child vision screening is undertaken. Cross-sectional studies have established an association between amblyopia and lower academic self-concept, slower reading speed. No difference has been found in educational performance in adolescence whilst there are mixed associations with educational attainment in adults. Educational trajectories and intentions have not been studied previously. We analyse if those treated for amblyopia have different educational performance and trajectories for core subjects during statutory schooling, or subsequent higher education (university) intentions than their peers without eye conditions.

**Methods and findings:**

Data from the Millennium Cohort Study of children born in the United Kingdom in 2000–01 and followed-up to age 17 years (*n* = 9989). Using a validated approach drawing on parental self-report on eye conditions and treatment coded by clinical reviewers, participants were grouped into mutually exclusive categories: no eye conditions, strabismus alone, refractive amblyopia, strabismic/mixed (refractive plus strabismic) amblyopia. The outcomes were levels and trajectories of passing English, Maths, Science at ages 7–16 years, passing national exams at age 16, and intentions at ages 14–17 to pursue higher (university) education. Adjusted analyses showed that amblyopia status was not associated with performance in English, Maths, and Science at any key stage, attainment in national exams, or intending to go to university. Similarly, the age-related trajectories of performance in core subjects and higher education intentions did not differ between the groups. There were no significant differences in main reason for having or not having university intentions.

**Conclusions:**

We found no associations between a history of amblyopia and either adverse performance or age-related attainment trajectories in core subjects during key stages of statutory schooling as well as the absence of an association with intentions for higher education. These results should be reassuring to affected children and young people, and their families, teachers and physicians.

## Introduction

Health and education are linked in a virtuous circle where children with better health tend to perform better at school, which in turn improves their wellbeing and enables them to achieve their full potential in education [1, 2]. Performance at key stages in primary and secondary schooling in turn is strongly associated with educational attainment, income, and health later in life [3, 4]. Children’s educational performance and aspirations are shaped by a number of factors including parental education, socioeconomic status, and expectations for their children [5–7]. Furthermore, parental involvement, expectations, and support during adolescence is positively associated with children’s career success, which is mediated partly by educational attainment [8].

Amblyopia is a neurodevelopmental condition resulting in reduced vision, predominantly affecting one eye, and occurs in up to 4% of children aged younger than 6 years in industrialised countries [1] Universal child vision screening exists in many countries with the primary aim of identifying children with amblyopia to implement treatment to improve vision as early as possible [9, 10]. There remains a limited evidence base on the broader impacts of amblyopia on affected individuals. Our previous research has shown no clinically meaningful differences in early cognitive performance of children with amblyopia compared to those without any eye conditions [11]. Other studies have reported lower academic self-concept and associated slower reading speed [12–14]. There is no indication of differences in educational performance between ages 7 and 18 years in older birth cohorts [15, 16]. Regarding educational attainment in adults, cross-sectional analyses provide evidence of mixed associations [16–19]. Educational trajectories of children with amblyopia and their expectations for further education, and whether and how these are related to each other have not been investigated.

We hypothesised that those who have been treated for amblyopia may experience educational impacts on discernible in longitudinal performance in core subjects at key stages during statutory schooling. Additionally, we hypothesised that there may be differences in intentions regarding higher education in adolescents who had been treated for amblyopia compared to those without any eye conditions. We investigated this analysing data of the Millennium Cohort Study (MCS) [20] to expand our previous investigation of school readiness and cognitive performance trajectories during early schooling in the same children [11].

## Materials and methods

### Study design

The Millennium Cohort Study is a national, prospective cohort study of children born in the United Kingdom (UK) in 2000–01 that used a stratified sampling design to achieve an overrepresentation of families from an ethnic minority group and deprived background [20]. Whilst this increased the absolute numbers of hard-to-reach groups, sampling weights were provided to ensure analyses are representative of the UK population throughout follow-up [21]. This renders it particularly suitable for research on amblyopia and educational outcomes, which vary by ethnicity and socioeconomic status [5, 22].

We built on our previous work [11] that identified children with eye conditions at age three to seven years, during which childhood vision screening is undertaken in the UK (ages four to five years) and thus all children with amblyopia would be expected to be identified with their treatment mostly complete [9]. We used this as the baseline for a longitudinal analysis to the end of statutory schooling (up to 17 years). Inclusion criteria were consent and successful linkage of education records from the National Pupil Database of England [23]. Children with neurological or -developmental conditions (such as cerebral palsy and Down syndrome), or eye conditions other than amblyopia or strabismus, were excluded as these conditions could themselves impact on both vision and educational outcomes [15, 24, 25]. Finally, children from multiple births were excluded as they are more likely to have vision disorders [24].

The MCS secured ethical approval from the National Health Service Multi-Centre Research Ethics Committee for each sweep (see https://cls.ucl.ac.uk/wp-content/uploads/2017/07/MCS-Ethical-Approval-and-Consent-2019.pdf). Written informed consent was required and obtained from parents/legal guardians. At the age 14 follow-up, children also provided informed consent. This study required no further ethical approval for secondary data analysis. All methods comply with the relevant national and institutional committees on human experimentation, including the ethical standards outlined in the 1975 Helsinki Declaration, as revised in 2013. The MCS was approved by the relevant Ethics Committees [20] and families of participants gave informed consent to participate [20]. Although the MCS is active in participant and public engagement, there was no direct involvement in this specific study, which drew on existing data.

### Ophthalmic data

We used our previously validated approach [11, 24, 25] drawing on the International Classifications of Diseases (ICD) and our extended taxonomy applied in our previous research on childhood blindness [26] to identify those with amblyopia from highly detailed parental report on their child’s vision and eye problems at ages 3, 5, and 7 years using validated open and closed-ended questions designed and reviewed by ophthalmologist in our study team. A conservative, hierarchical approach was followed thus requiring diagnosis, types of treatment and age at treatment to match consistently. The therapies included surgery, patch occlusion or penalisation with cycloplegic drops, and glasses.

In this follow-up study, we categorised children in the same mutually exclusive groups that we have used in prior research [11], comprising two ‘affected’ groups of a) refractive amblyopia and b) strabismic or mixed amblyopia along with two comparator groups of c) no eye conditions (main reference) and d) strabismus alone, the latter to differentiate between the impact of impaired acuity (amblyopia) and ocular misalignment *per se*. Refractive amblyopia has the best prognosis of all amblyopia types, impacting mostly acuity, whereas strabismic amblyopia directly affects stereovision as well, such that mixed amblyopia is clinically the most ‘severe’ type.

### Education data

In England, the National Curriculum consists of five Key Stages (KS) [27]. All pupils must attend full-time education until the end of Key Stage 4. At that time, pupils take a series of exams called the General Certificate of Secondary Education (GCSE). The educational records at Key Stages 1, 2, and 4 were linked to Millennium Cohort Study members, with tests’ results at the end of each stage corresponding to ages 7, 11, and 16 years, respectively [23]. In the mandatory subjects English (reading and writing), mathematics, and science, pupils are expected to achieve at least level two at the end of Key Stage 1 and level four at Key Stage 2. At the end of Key Stage 4, pupils are expected to achieve at least five GCSEs A*-C, including English and mathematics. We used these benchmarks as our outcomes.

Our secondary outcome of interest was adolescents’ views at ages 14 and 17 years of how likely they were to go to university on a scale of 0 to 100%. As this continuous variable has a bounded range and is non-normally distributed, its values were dichotomised to ‘present’ if ≥median score and ‘absent’ if <median. Additionally, participants’ main reason at age 17 years about going or not going to university were analysed for context. See S1 Table in the Supplement for detailed coding of outcomes.

### Confounders/covariates

Due to the sociodemographic patterning in amblyopia risk and educational outcomes, important confounders at baseline were sex, ethnicity, preterm birth, maternal education, household income [5, 11, 15–17]. Possible confounders for educational trajectories [5, 15, 28, 29] included special education needs (SEN) for any reason including vision (at each Key Stage), academic self-concept as the level of agreement in being good at the mandatory subjects (ages 11 and 14), and parents’ expectations for their child to go to university (ages 14 and 17). See S1 Table for detailed coding of covariates.

### Statistical analyses

Analyses were conducted using R version 4.1.0 [30]. Sampling weights were used to adjust for the Millennium Cohort Study survey design and attrition over time [21]. Logistic regression models were fitted to understand potential selection bias due to non-consent to data linkage of education results and missing data in university intentions [31]. Non-consent in the outcomes was dealt with by pairwise deletion to maximise the sample size for each outcome of interest. Of the cohort, 27% did not have linked educational records due to no consent for linkage or moving abroad, which resulted in a study sample with higher representation of those from other than white backgrounds and more affluent families (S2 Table). There were 28% missing observations of university intentions with higher odds of missing intentions among those from lower educated, poorer families or who failed their GCSE (S2 Table). This supports the hypothesis that those who had missing university intentions were either unlikely to plan to go to university or were uncertain about going. Therefore, missingness in such intentions was informative [31] and coded as ‘absent’. As a sensitivity analysis, a complete-case analysis was also performed (i.e. excluding those with missing university intentions). Missing data were minimal in covariates (<2%), therefore these subjects were excluded from analyses [32].

Chi-squared tests assessed differences by amblyopia status in covariates and reasons for university intentions. Logistic regression models of passing English, Maths, and Science at each Key Stage, passing GCSE at Key Stage 4, and having university intentions at ages 14 and 17 were fitted to assess associations with amblyopia. Fixed-effects models assessed the performance levels of educational outcomes, whereas mixed-effects models assessed their age-related trajectories and included a random effect on participant to account for repeated measures. The models were adjusted for all baseline covariates and those educational covariates (i.e. special education needs, academic-self concept, and parents’ university expectations) that were significant at *α* = 0.05 to obtain parsimonious models. Additionally, significant interactions between amblyopia and sex, age, and parents’ university expectations were included in the final models. Collinearity was assessed via variance inflation factors. All models’ assumptions were satisfied. In our previous study [11] we did not find a mediating effect of age of treatment on school readiness or early cognitive trajectories, therefore treatment was not considered here.

## Results

### Study population

The cohort consisted of 9989 singletons aged seven years old who were followed-up to age 17 (Table 1). The proportion of the study sample with a history of refractive amblyopia was 7 per 1000 children, 21 per 1000 for strabismic or mixed amblyopia and 28 per 1000 for strabismus alone. The proportions varied significantly by preterm birth and socioeconomic background, as well as with a history of special education needs and parents’ university expectations (Table 2). There were no differences in finding oneself to be good at English, mathematics, and science by amblyopia status (results not shown to avoid potential statistical disclosure), thus these were not adjusted for in the regression models for educational trajectories and university intentions.

**Table 1:** Study population of singletons.

Excluded	MCS sweep	Linked educational record	University intentions
Total, *n* = 431 Other eye conditions than amblyopia and strabismus (*n* = 120) Neurological or -developmental conditions (*n* = 65) Missing baseline characteristics (*n* = 195)	MCS4 ~7 years *n* = 9989 (100%)	KS1 *n* = 7321 (73%)	
Attrition *n* = 1235 (12%)	MCS5 ~11 years *n* = 8754 (88%)	KS2 *n* = 7225 (72%)	
Attrition *n* = 872 (9%)	MCS6 ~14 years *n* = 7882 (79%)		*n* = 7882 (79%)
Attrition *n* = 675 (7%)	MCS7 ~17 years *n* = 7207 (72%)	KS4 *n* = 7415 (74%)	*n* = 7207 (72%)

KS, Key Stage; MCS, Millennium Cohort Study.

**Table 2:** Baseline and education characteristics of study population by history of amblyopia and/or strabismus at age 7 years.

Covariate		Category	No eye condition (weighted %)	Strabismus alone (weighted %)	Refractive amblyopia (weighted %)	Strabismic or mixed amblyopia (weighted %)	χ^2^ (weighted) *p*-value^a^
**Baseline *(observed/unweighted n = 9989)***							
**Sex**		Boys	4777 (51)	141 (51)	100 (53)	39 (58)	0.752
		Girls	4635 (49)	144 (49)	106 (47)	29 (42)	
**Preterm birth**		No	8829 (94)	247 (86)	182 (89)	^b^	**<0.001**
		Yes	601 (6)	38 (14)	24 (11)	^b^	
**Maternal education**		A-levels or higher	3316 (34)	80 (27)	59 (25)	16 (23)	**<0.001**
		O-levels	3240 (36)	107 (36)	62 (30)	21 (33)	
		None	2874 (30)	98 (37)	85 (45)	31 (44)	
**Household income quintile**		1 Richest	1953 (21)	44 (16)	35 (17)	^b^	0.141
		2	1850 (20)	56 (20)	34 (15)	^b^	
		3	1873 (20)	66 (24)	37 (20)	^b^	
		4	1822 (19)	61 (21)	49 (24)	^b^	
		5 Poorest	1932 (20)	58 (19)	51 (24)	^b^	
**Education *(observed/unweighted n variable due to attrition and linked education records*, *ranging between 5820–7491)***							
**Special education needs (SEN) history**	Key Stage 1	No	5685 (81)	127 (65)	124 (76)	41 (80)	**<0.001**
		Yes	1334 (19)	68 (35)	39 (24)	10 (20)	
	Key Stage 2	Same	5098 (73)	105 (54)	109 (67)	34 (67)	**<0.001**
		Became SEN	1921 (27)	90 (46)	54 (33)	17 (33)	
	Key Stage 4	Same	4881 (70)	98 (50)	98 (60)	31 (61)	**<0.001**
		Became SEN	2138 (30)	97 (50)	65 (40)	20 (39)	
**Parents’ university expectations for child**	Age 14	Unlikely	1598 (22)	74 (37)	40 (30)	19 (35)	**<0.001**
		Likely	5505 (78)	126 (63)	94 (70)	35 (65)	
	Age 14 → 17	No longer likely	1385 (25)	32 (21)	30 (29)	12 (28)	0.537
		Same/likely	4135 (75)	120 (79)	75 (71)	31 (72)	

^a^ *p*<0.05 in **bold**;

^b^ Not provided to avoid potential disclosure.

### Key stage performance levels and trajectories (Table 3 and S3–S6 Tables)

**Table 3:** Adjusted odds ratios of subject-specific Key Stage (KS) attainment levels and age-related trajectories, GCSE attainment, and university intentions.

Outcome^a^	Eye status	KS1 aOR (95%CI)	KS2 aOR (95%CI)	KS4 aOR (95%CI)	Across KS aOR (95%CI)
**English (per KS, *n* = 6972)**	No eye condition	1.00	1.00	1.00	1.00
	Strabismus alone	0.71 (0.47–1.07)	0.78 (0.52–1.20)	1.16 (0.81–1.67)	0.91 (0.73–1.15)
	Refractive amblyopia	1.10 (0.69–1.80)	0.75 (0.47–1.24)	0.88 (0.61–1.29)	1.04 (0.80–1.36)
	Strabismic/mixed amblyopia	0.61 (0.29–1.38)	1.03 (0.43–2.78)	1.28 (0.64–2.60)	1.00 (0.63–1.60)
**Mathematics (per KS, *n* = 6989)**	No eye condition	1.00	1.00	1.00	1.00
	Strabismus alone	0.75 (0.47–1.23)	0.78 (0.52–1.20)	1.02 (0.72–1.45)	0.86 (0.68–1.10)
	Refractive amblyopia	1.03 (0.57–1.96)	0.75 (0.47–1.24)	0.86 (0.59–1.26)	0.86 (0.66–1.13)
	Strabismic/mixed amblyopia	0.54 (0.21–1.62)	1.03 (0.43–2.78)	0.95 (0.48–1.93)	0.87 (0.54–1.44)
**Science (per KS, *n* = 6935)**	No eye condition	1.00	1.00	1.00	1.00
	Strabismus alone	0.74 (0.47–1.19)	0.79 (0.51–1.25)	0.87 (0.61–1.25)	0.81 (0.64–1.03)
	Refractive amblyopia	1.23 (0.69–2.33)	0.65 (0.39–1.11)	0.78 (0.54–1.14)	0.84 (0.64–1.10)
	Strabismic/mixed amblyopia	0.66 (0.26–1.93)	0.59 (0.25–1.54)	1.25 (0.63–2.55)	0.89 (0.55–1.47)
**GCSE (*n* = 7422)**	No eye condition			1.00	
	Strabismus alone			1.07 (0.76–1.49)	
	Refractive amblyopia			0.73 (0.51–1.06)	
	Strabismic/mixed amblyopia			1.52 (0.79–2.97)	
**University intentions (per KS, *n* = 6581)**	No eye condition		1.00	1.00	1.00
	Strabismus alone		0.84 (0.54–1.30)	1.14 (0.69–1.85)	0.95 (0.68–1.31)
	Refractive amblyopia		1.06 (0.66–1.68)	0.79 (0.42–1.42)	0.93 (0.64–1.33)
	Strabismic/mixed amblyopia		1.14 (0.47–2.70)	0.69 (0.22–1.95)	0.92 (0.46–1.78)

^a^ Independent outcomes with their own regression model. All odds ratios (OR) adjusted for sex, ethnicity, preterm birth, maternal education, household income, special education needs, and survey weights. Age-related trajectories in English, mathematics and science across Key Stages (KS) additionally adjusted for age and multi-level on participant. Age-related trajectories in university intentions additionally adjusted for age, English and mathematics performances, and parents’ university expectations, and multi-level on participant. *P*<0.05 in **bold**. All variance inflation factors were between 1.01 and 1.92, indicating no multicollinearity.

In order of decreasing effect size, the following factors were significantly associated with lower performance/attainments (S3–S6 Tables): having special education needs, lower maternal education level, and lower household income. Furthermore, boys performed significantly lower in English and overall GCSE, whereas girls in mathematics. Children from an ethnic minority background did not perform significantly worse than those from a white ethnic background, in fact those from black/African/Caribbean or South Asian background performed better in English, science and GCSE, and those from a South Asian background additionally in mathematics.

There were no significant differences in the performance levels of English, mathematics, and science at any given Key Stage between those treated for amblyopia and those without eye conditions status (*p*-values range, 0.228–0.883), nor any differences in performance level trajectories of these core subjects across Key Stages (0.208–0.636) (adjusted OR’s presented in Table 3). Similarly, GCSE performance at the end of statutory schooling did not differ by amblyopia status (*p*, 0.212) (Table 3). This absence of an association between amblyopia status and performance of core subjects and GCSE was the same for boys and girls (0.236–0.904).

### University intentions, trajectories, and reasons (Table 3 and S7 Table)

At ages 14 and 17 years, half of study participants reported that thought they were 80% and 75%, respectively, likely to go to university. Participants reporting these probabilities or higher were categorised as intending to go to university. The factors most strongly associated with university intention of an adolescent were their parents’ expectations that they were likely to do so (adjusted OR, 7.75; 95%CI, 6.68–9.02) and, in order of decreasing effect size, maternal education, sex, special education needs, ethnicity, English and mathematics performances, household income, and age (S7 Table).

There were no significant differences by between those treated for amblyopia and those without eye conditions in university intention at ages 14 and 17 years (*p*, 0.860 and 0.717, respectively) nor were there any differences in their age-related trajectories (0.959) (Table 3). These results were the same when split by sex (0.377) or parents’ university expectations for their child (0.405).

To test the sensitivity of the results in handling the missing data, a complete-case analysis was carried out, which estimated very similar aOR and their standard errors of university intentions associated with amblyopia status. The conclusions of no significant difference did not change (S8 Table).

**Fig 1:**
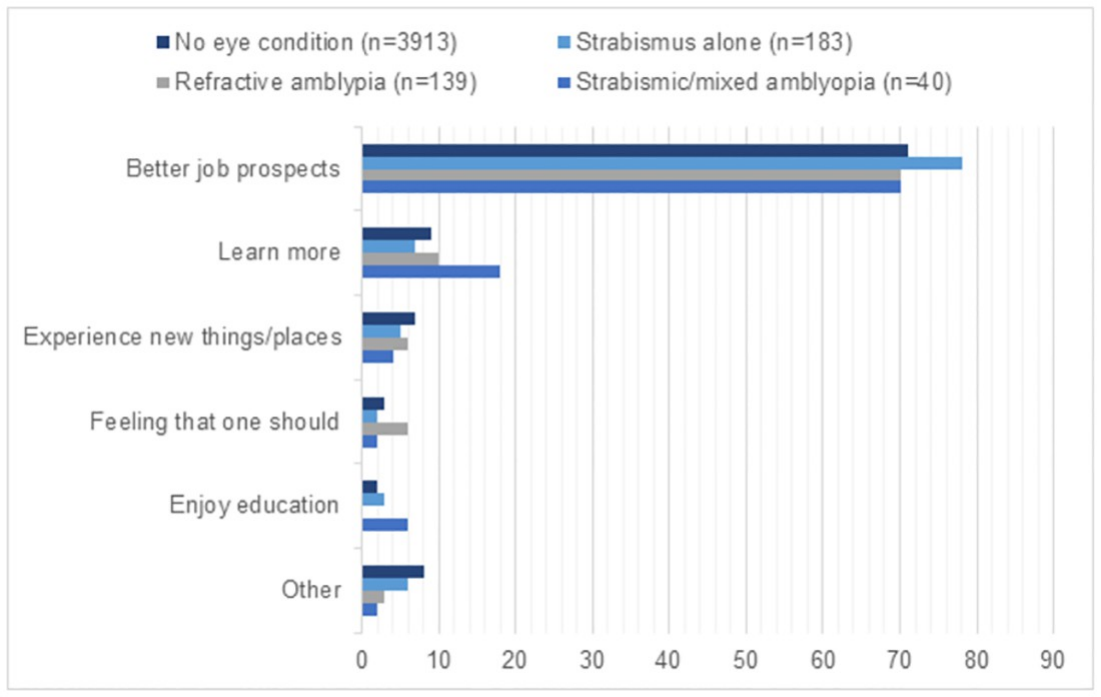
17-year-olds’ main reason why likely to go to university by amblyopia and/or strabismus status (*n* = 4275). Weighted percentages presented. Category ‘other’ included reasons of family recommends it, teachers recommend it, enjoy social life, get away from home, and other. No one reported as main reason because friends will go. There were no significant differences in reason for having university intentions by amblyopia and/or strabismus status (*p* = 0.966).

**Fig 2:**
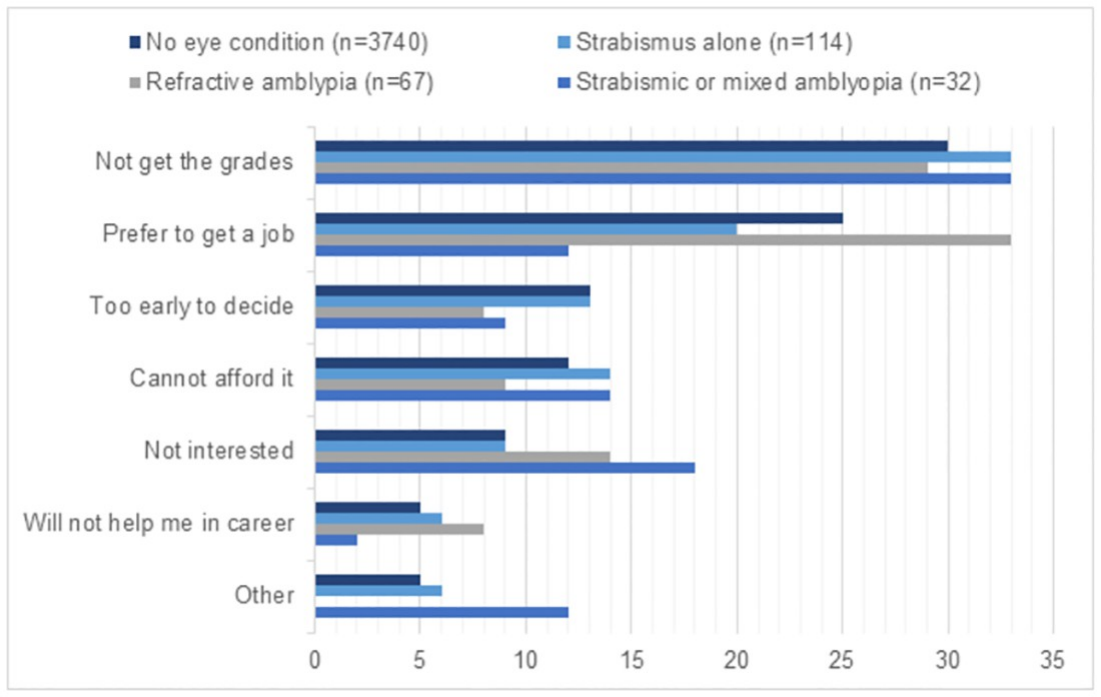
17-year-olds’ main reason why unlikely to go to university by amblyopia and/or strabismus (*n* = 3953). Weighted percentages presented. Category ‘other’ included reasons of starting a family and other. No one reported as main reason because family recommends it and friends plan to leave. There were no significant differences in reason for having no university intentions by amblyopia and/or strabismus status (*p* = 0.780).

Those treated for amblyopia did not differ from those without eye conditions in the main reason for reporting they were or were not likely to attend university (*p*, 0.966 and 0.780, respectively). The most common reasons for intending to go to university were better job prospects (71%) and learning more (9%) (Fig 1). The most common reasons for *not* intending to go to university were being likely not to get the school exam grades required (30%), followed by preferring to get a job (25%), feeling it was too early to decide (13%), and not being able to afford it (12%) (Fig 2).

## Discussion

From a population-based cohort study, no differences were found between children treated for amblyopia and those without any eye conditions in performance levels or trajectories of performance in the core subjects from age 7 years and across the key stages of statutory schooling in the UK. There were also no differences by amblyopia status in overall performance at the end of statutory schooling nor in the subsequent plans about going to university and the underlying reasons for their choices.

The Millennium Cohort Study provided particular advantages for this investigation because of its overall size and over-sampling of subgroups known to be at greater risk of both amblyopia and strabismus [22] and poor educational outcomes [5]. Performance throughout compulsory schooling were observed in the same children, enabling, our novel investigation of trajectories. Self- and parent-reported university intentions allowed investigation of both parties’ perceived impact of prior history of amblyopia on education given objective educational performance, and how these three interrelate. Restriction of the study sample by excluding those with eye conditions other than amblyopia or strabismus also with neurological or -developmental disorders permitted a specific investigation of the impact of amblyopia status.

Study limitations include the possibility of misclassification of amblyopia and strabismus based on parental reports of eye conditions and treatment, although this risk is low, with prior validation and application of this approach [11, 24, 25], including age at treatment initiation by type of amblyopia following clinical expectations in a consistent pattern [11], and as supported by the proportions of amblyopia and strabismus found in this study which align closely with similar population studies in the UK [22]. By definition, all participants with amblyopia had received treatment–therefore our study does not address whether there is an association between undiagnosed and therefore untreated amblyopia and educational outcomes, which is an important question. Bias through not consenting to educational data linkage resulted in a study sample with higher representation of those from other than white backgrounds and more affluent families. Attrition and missing data are common problems in cohort studies. Attrition was handled using sample weights [21] in this study, whereas missing observations in baseline characteristics were negligible and missing observations for university intentions were thoroughly examined by descriptive and regression analyses, and appropriately dealt with by combining it to the informed category and carrying out a sensitivity analysis [31]. Although regression models were adjusted for key known confounders [5, 11, 15–17, 28, 29], as in any observational study potential residual confounding cannot be ruled out. Regression models across key stages were more robust than those at a specific key stage, as evidenced by the width of the confidence interval, yet moderate associations if present would have been picked up by the longitudinal models, therefore our conclusions still hold. The Millennium Cohort Study does not contain data on teachers’ expectations for their pupils to attend university or any career counselling offered. Finally, as our study investigated associations at the population rather than individual level (as is appropriate for considering the benefits of a universal population screening programme), we are reporting associations ‘on average’ and as such it is possible that some pupils with amblyopia had adverse or positive educational outcomes that are not captured in the summary statistics.

There are no directly comparable longitudinal studies with which to compare our findings. However our finding of an absence of association between a history of treatment for either amblyopia or strabismus and educational achievements in core subjects from age 7 years onwards aligns with our previous research on early schooling up to age 7 years in the same individuals [11] as well as with other cross-sectional studies of educational performance between ages 7 and 16 years [15, 16].

Studies investigating associations between amblyopia and diverse outcomes into adult life have conflicting findings: an association with lower lifetime educational attainment has been reported in older [18, 19] rather than more recent cohorts [16–18], which is consistent with changing mitigating factors rather than a change in the direct impact of amblyopia itself. Our contemporary cohort showed, reassuringly, that neither attainment itself nor intentions of further education (university) differed by amblyopia status. It would be reasonable to expect therefore that educational attainment later in life would not differ. These findings should be encouraging to current and future populations of affected children and their parents. Despite prior studies reporting that children with amblyopia or strabismus are more likely to be bullied [33, 34] and have lower scholastic self-perception [12], it is important to note that within our contemporary cohort, those treated for amblyopia did not have lower confidence on average in their performance in core subjects nor differ in their assessment of their prospects for further education than those without any eye conditions. Parental expectations highly influence children’s intentions regarding higher education [5–8] but we found no synergistic effect between parents expectations for their children and their child’s amblyopia status, which is also encouraging.

## Conclusions

Our study found no association between having been treated for amblyopia and educational performance during statutory schooling nor intentions for further education. These findings should reassure families, teachers, and clinicians and policy makers that having amblyopia need not be considered a barrier to educational outcomes or ambitions.

## Supporting information

S1 Checklist STROBE statement—Checklist of items that should be included in reports of *cohort studies*. (DOCX)

S1 Table Coding of covariates and outcomes. (DOCX)

S2 Table Adjusted odds of non-consent education data linkage and missing data in university intentions (*n =* 9989). (DOCX)

S3 Table Trajectories of achieving Key Stage (KS) levels of English. (DOCX)

S4 Table Trajectories of achieving Key Stage (KS) levels of mathematics. (DOCX)

S5 Table Trajectories of achieving Key Stage (KS) levels of science. (DOCX)

S6 Table Adjusted effects associated with passing GCSE (*n =* 7422). (DOCX)

S7 Table Trajectories of adolescents’ university intentions. (DOCX)

S8 Table Complete case analysis of adolescents’ university intentions. (DOCX)
