# Supplementary material for: Educational attainment and trajectories at key stages of schooling for children with amblyopia compared to those without eye conditions: Findings from the Millennium Cohort Study
Source: PLoS One. 2023 Mar 30;18(3):e0283786. doi: 10.1371/journal.pone.0283786 (PMC10062655; doi:10.1371/journal.pone.0283786)
Supplement: S1 Table — (DOCX) [file pone.0283786.s002.docx]

**Table S1. Coding of covariates and outcomes.**

| Concept | Description | Coding |
| --- | --- | --- |
| *Covariate* | | |
| Academic self-concept | Responses to “I am good at” for the subjects English, Maths, and Science, measured at survey sweeps 5-6 (child aged 11 or 14 Years). | Per subject: Strongly agree (reference) / agree / disagree or strongly disagree.  Age-related change: higher level of agreement / same (reference) / lower level of agreement |
| Amblyopia and/or strabismus status | Case definition: at least one parental report of amblyopia and/or strabismus and at least one report on related treatment (surgery, occlusion by patch or penalisation using cycloplegic drops, and/or spectacles) measured at survey sweeps 2-4 (child aged 3, 5, or 7 years). | No eye condition (reference) / strabismus alone / refractive amblyopia / strabismic or mixed amblyopia. |
| Ethnicity | Ethnicity of child, measured at survey sweep 4 (child aged 7 years). | Black, African, Caribbean / South Asian / White (reference) / Other. |
| Eye conditions | Eye conditions other than amblyopia and strabismus, nasolacrimal abnormalities, superficial infections, dry eyes, and anisocoria, measured at survey sweep 2-4 (child aged 3, 5, or 7 years). Children with eye conditions were excluded from the analyses. | No (reference) / yes. |
| Household income | Annual disposable household income in quintiles, measured at survey sweep 4 (child aged 7 years). | Highest (1, reference) to lowest (5). |
| Maternal education | Highest obtained qualification by mother of child, measured at survey sweep 4 (child aged 7 years). | Higher degree, diploma, or A-levels (reference) / O-levels or other / no qualifications. |
| Neurological conditions | All ICD10 G-codes except for G43 migraine, G44 headache, and G47 sleep disorder, measured at survey sweep 4 (child aged 7 years). Children with neurological conditions were excluded from the analyses. | No (reference) / yes. |
| Parental university expectations | Parental response to “How likely or unlikely do you think it is that your child will attend university?” measured at survey sweeps 6-7 (child aged 14 or 17 years). | Unlikely (*not at all likely, not very likely, or NA;* reference) / likely (*fairly likely* / *very likely*). |
| Preterm birth | Child born before 37 weeks of gestational age, measured at survey sweep 1 (child aged 9 months). | No (reference) / yes. |
| Sex | Sex of child, measured at survey sweep 4 (child aged 7 years). | Boy (reference) / girl. |
| Special education needs | Special education needs (SEN), measured at Key Stages 1, 2, and 4 (child aged 7, 11, and 16 years). | No (reference) / yes. |
| University intentions – why no | Self-report of the main reason why cohort member might or will not go to university, measured at survey sweep 7 (child aged 17 years). | Not get the grades (reference) / Prefer to get a job / Too early to decide / Cannot afford it / Not interested / Will not help me in career / Other (including starting a family, family recommends to leave, friends plan to leave, and other) |
| University intensions – why yes | Self-report of the main reason why cohort member might or will go to university, measured at survey sweep 7 (child aged 17 years). | Better job prospects (reference) / Learn more / Experience new things/places / Feeling that one should / Enjoy education / Other (including family recommends it, teachers recommend it, friends will go, enjoy social life, get away from home, and other) |
| *Outcome* | | |
| GCSE | Achieving national curriculum of five GCSEs A*-C, including English and mathematics, measured at age 16 years. | No (reference) / yes. |
| KS English | Achieving national curriculum level of two and four at end of Key Stage 1 and 2, respectively, and grade A*-C at the end of Key Stage 4 (child aged 7, 11, and 16 years). | No (reference) / yes. |
| KS maths | Achieving national curriculum level of two and four at end of Key Stage 1 and 2, respectively, and grade A*-C at the end of Key Stage 4 (child aged 7, 11, and 16 years). | No (reference) / yes. |
| KS science | Achieving national curriculum level of two and four at end of Key Stage 1 and 2, respectively, and grade A*-C at the end of Key Stage 4 (child aged 7, 11, and 16 years). | No (reference) / yes. |
| University – cohort member’s intentions | Cohort member’s response to “How likely or unlikely do you think you will attend university?” measured at survey sweeps 6-7 (child aged 14 and 17 years). | Absent (*%≤median or NA;* reference) / present (*%>median*). |
