# Supplementary material for: Educational attainment and trajectories at key stages of schooling for children with amblyopia compared to those without eye conditions: Findings from the Millennium Cohort Study
Source: PLoS One. 2023 Mar 30;18(3):e0283786. doi: 10.1371/journal.pone.0283786 (PMC10062655; doi:10.1371/journal.pone.0283786)
Supplement: S2 Table — (DOCX) [file pone.0283786.s003.docx]

**Table S2. Adjusted odds of non-consent education data linkage and missing data in university intentions (*n=*9989).**

| Covariate | Category | Non-consent education  data linkage aOR (95%CI)^a,b^ | Missing university intentions aOR (95%CI)^a,c^ |
| --- | --- | --- | --- |
| Eye condition | No eye condition | 1.00 | 1.00 |
|  | Strabismus alone | 0.99 (0.69-1.38) | 0.97 (0.72-1.31) |
|  | Refractive amblyopia | 0.74 (0.52-1.03) | 1.25 (0.89-1.76) |
|  | Strabismic/mixed amblyopia | 1.03 (0.52-1.92) | 0.96 (0.54-1.72) |
| Sex | Boys | 1.00 | 1.00 |
|  | Girls | 1.03 (0.92-1.16) | **0.67 (0.61-0.73)** |
| Ethnicity | Black, African, Caribbean | **0.48 (0.34-0.67)** | 1.10 (0.86-1.40) |
|  | South Asian | **0.31 (0.25-0.39)** | 0.87 (0.75-1.02) |
|  | White | 1.00 | 1.00 |
|  | Other | **0.46 (0.34-0.62)** | 0.95 (0.76-1.18) |
| Preterm birth | No | 1.00 | 1.00 |
|  | Yes | 1.16 (0.93-1.44) | 1.01 (0.83-1.22) |
| Maternal education | A-levels or higher | 1.00 | 1.00 |
|  | O-levels | 0.87 (0.75-1.00) | **1.58 (1.40-1.78)** |
|  | None | 0.95 (0.81-1.12) | **1.91 (1.66-2.19)** |
| Household income quintile | 1 Richest | 1.00 | 1.00 |
|  | 2 | 1.15 (0.97-1.37) | 1.12 (0.97-1.30) |
|  | 3 | **1.32 (1.09-1.58)** | **1.23 (1.06-1.44)** |
|  | 4 | **1.58 (1.30-1.93)** | **1.39 (1.18-1.64)** |
|  | 5 Poorest | **1.81 (1.48-2.23)** | **1.61 (1.36-1.92)** |
| University intentions | Unlikely | 1.00 | 1.00 |
|  | Likely | 1.06 (0.93-1.20) |  |
| Key Stage achieved | No |  |  |
|  | Yes |  | **0.57 (0.51-0.63)** |

^a^ Odds ratios adjusted (aOR) for all covariates listed in the table and sample weights; *p*<0.05 in **bold**.
^b^ 2574 (26%) missing.
^c^ 2782 (28%) missing.
