# Supplementary material for: Educational attainment and trajectories at key stages of schooling for children with amblyopia compared to those without eye conditions: Findings from the Millennium Cohort Study
Source: PLoS One. 2023 Mar 30;18(3):e0283786. doi: 10.1371/journal.pone.0283786 (PMC10062655; doi:10.1371/journal.pone.0283786)
Supplement: S3 Table — (DOCX) [file pone.0283786.s004.docx]

**Table S3. Trajectories of achieving Key Stage (KS) levels of English.**

| **Covariate** | **Category** | **KS1 (*n=*6972)**  **aOR (95%CI)** | **KS2 (*n=*6972)**  **aOR (95%CI)** | **KS4 (*n=*6972)**  **aOR (95%CI)** | **Across KS (*n=*20,916)**  **aOR (95%CI)** |
| --- | --- | --- | --- | --- | --- |
| Eye status | No eye condition | 1.00 | 1.00 | 1.00 | 1.00 |
|  | Strabismus alone | 0.71 (0.47-1.07) | 0.78 (0.52-1.20) | 1.16 (0.81-1.67) | 0.91 (0.73-1.15) |
|  | Refractive amblyopia | 1.10 (0.69-1.80) | 0.75 (0.47-1.24) | 0.88 (0.61-1.29) | 1.04 (0.80-1.36) |
|  | Strabismic/mixed amblyopia | 0.61 (0.29-1.38) | 1.03 (0.43-2.78) | 1.28 (0.64-2.60) | 1.00 (0.63-1.60) |
| Sex | Boys | 1.00 | 1.00 | 1.00 | 1.00 |
|  | Girls | **1.43 (1.23-1.66)** | **0.58 (0.49-0.68)** | **1.90 (1.69-2.14)** | **1.62 (1.49-1.79)** |
| Ethnicity | Black/African/Caribbean | 0.92 (0.66-1.30) | **1.70 (1.16-2.57)** | **1.85 (1.39-2.50)** | **1.56 (1.29-1.91)** |
|  | South Asian | 1.10 (0.89-1.37) | 0.95 (0.75-1.21) | **2.17 (1.79-2.63)** | **1.51 (1.34-1.72)** |
|  | White | 1.00 | 1.00 | 1.00 | 1.00 |
|  | Other | 1.30 (0.93-1.84) | 1.14 (0.80-1.66) | **1.65 (1.26-2.20)** | **1.55 (1.28-1.89)** |
| Preterm birth | No | 1.00 | 1.00 | 1.00 | 1.00 |
|  | Yes | **0.69 (0.53-0.91)** | 0.76 (0.57-1.01) | 0.90 (0.72-1.14) | **0.81 (0.69-0.94)** |
| Maternal education | A-levels or higher | 1.00 | 1.00 | 1.00 | 1.00 |
|  | O-levels | **0.65 (0.52-0.80)** | **0.54 (0.42-0.69)** | **0.66 (0.57-0.78)** | **0.66 (0.59-0.74)** |
|  | None | **0.39 (0.31-0.48)** | **0.41 (0.32-0.53)** | **0.42 (0.36-0.50)** | **0.41 (0.36-0.46)** |
| Household income quintile | 1 Richest | 1.00 | 1.00 | 1.00 | 1.00 |
|  | 2 | 0.87 (0.65-1.18) | 0.80 (0.57-1.10) | 1.00 (0.81-1.24) | 0.93 (0.79-1.08) |
|  | 3 | **0.65 (0.49-0.87)** | **0.70 (0.51-0.97)** | **0.73 (0.59-0.90)** | **0.70 (0.60-0.82)** |
|  | 4 | **0.56 (0.41-0.74)** | **0.65 (0.47-0.89)** | **0.52 (0.42-0.64)** | **0.57 (0.49-0.66)** |
|  | 5 Poorest | **0.38 (0.29-0.51)** | **0.66 (0.48-0.91)** | **0.44 (0.36-0.55)** | **0.46 (0.39-0.54)** |
| History of SEN at KS | No | 1.00 | 1.00 | 1.00 | 1.00 |
|  | Yes | **0.08 (0.07-0.09)** | **0.09 (0.08-0.11)** | **0.19 (0.17-0.21)** | **0.12 (0.11-0.13)** |
| Age | KS1 |  |  |  | 1.00 |
|  | KS2 |  |  |  | **2.91 (2.60-3.26)** |
|  | KS3 |  |  |  | **0.58 (0.53-0.64)** |

Odds ratios adjusted (aOR) for all covariates listed in the table and sample weights; *p*<0.05 in **bold**.
