# Supplementary material for: Educational attainment and trajectories at key stages of schooling for children with amblyopia compared to those without eye conditions: Findings from the Millennium Cohort Study
Source: PLoS One. 2023 Mar 30;18(3):e0283786. doi: 10.1371/journal.pone.0283786 (PMC10062655; doi:10.1371/journal.pone.0283786)
Supplement: S4 Table — (DOCX) [file pone.0283786.s005.docx]

**Table S4. Trajectories of achieving Key Stage (KS) levels of mathematics.**

| **Covariate** | **Category** | **KS1 (*n=*6989)**  **aOR (95%CI)** | **KS2 (*n=*6989)**  **aOR (95%CI)** | **KS4 (*n=*6989)**  **aOR (95%CI)** | **Across KS (*n=*20,967)**  **aOR (95%CI)** |
| --- | --- | --- | --- | --- | --- |
| Eye status | No eye condition | 1.00 | 1.00 | 1.00 | 1.00 |
|  | Strabismus alone | 0.75 (0.47-1.23) | 0.78 (0.52-1.20) | 1.02 (0.72-1.45) | 0.86 (0.68-1.10) |
|  | Refractive amblyopia | 1.03 (0.57-1.96) | 0.75 (0.47-1.24) | 0.86 (0.59-1.26) | 0.86 (0.66-1.13) |
|  | Strabismic/mixed amblyopia | 0.54 (0.21-1.62) | 1.03 (0.43-2.78) | 0.95 (0.48-1.93) | 0.87 (0.54-1.44) |
| Sex | Boys | 1.00 | 1.00 | 1.00 | 1.00 |
|  | Girls | 0.88 (0.72-1.07) | **0.58 (0.49-0.68)** | **0.86 (0.76-0.97)** | **0.77 (0.71-0.84)** |
| Ethnicity | Black/African/Caribbean | 0.72 (0.48-1.10) | **1.70 (1.16-2.57)** | 1.21 (0.92-1.62) | 1.20 (0.98-1.47) |
|  | South Asian | 0.93 (0.71-1.23) | 0.95 (0.75-1.21) | **1.61 (1.34-1.94)** | **1.22 (1.08-1.39)** |
|  | White | 1.00 | 1.00 | 1.00 | 1.00 |
|  | Other | 1.11 (0.73-1.76) | 1.14 (0.80-1.66) | 1.25 (0.96-1.64) | 1.19 (0.98-1.44) |
| Preterm birth | No | 1.00 | 1.00 | 1.00 | 1.00 |
|  | Yes | 0.86 (0.61-1.22) | 0.76 (0.57-1.01) | 1.04 (0.83-1.31) | 0.90 (0.77-1.06) |
| Maternal education | A-levels or higher | 1.00 | 1.00 | 1.00 | 1.00 |
|  | O-levels | **0.61 (0.44-0.84)** | **0.54 (0.42-0.69)** | **0.58 (0.50-0.68)** | **0.57 (0.50-0.65)** |
|  | None | **0.34 (0.25-0.47)** | **0.41 (0.32-0.53)** | **0.38 (0.32-0.45)** | **0.38 (0.33-0.43)** |
| Household income quintile | 1 Richest | 1.00 | 1.00 | 1.00 | 1.00 |
|  | 2 | 0.92 (0.59-1.40) | 0.80 (0.57-1.10) | 1.03 (0.83-1.27) | 0.94 (0.80-1.11) |
|  | 3 | 0.89 (0.58-1.34) | **0.70 (0.51-0.97)** | **0.80 (0.65-0.99)** | **0.78 (0.67-0.92)** |
|  | 4 | 0.76 (0.50-1.14) | **0.65 (0.47-0.89)** | **0.59 (0.48-0.73)** | **0.63 (0.54-0.74)** |
|  | 5 Poorest | **0.61 (0.40-0.91)** | **0.66 (0.48-0.91)** | **0.46 (0.37-0.57)** | **0.54 (0.46-0.63)** |
| History of SEN at KS | No | 1.00 | 1.00 | 1.00 | 1.00 |
|  | Yes | **0.07 (0.06-0.09)** | **0.09 (0.08-0.11)** | **0.18 (0.16-0.21)** | **0.13 (0.12-0.14)** |
| Age | KS1 |  |  |  | 1.00 |
|  | KS2 |  |  |  | **0.75 (0.67-0.85)** |
|  | KS3 |  |  |  | **0.21 (0.19-0.23)** |

Odds ratios adjusted (aOR) for all covariates listed in the table and sample weights; *p*<0.05 in **bold**.
