# Supplementary material for: Educational attainment and trajectories at key stages of schooling for children with amblyopia compared to those without eye conditions: Findings from the Millennium Cohort Study
Source: PLoS One. 2023 Mar 30;18(3):e0283786. doi: 10.1371/journal.pone.0283786 (PMC10062655; doi:10.1371/journal.pone.0283786)
Supplement: S5 Table — (DOCX) [file pone.0283786.s006.docx]

**Table S5. Trajectories of achieving Key Stage (KS) levels of science.**

| **Covariate** | **Category** | **KS1 (*n=*6935)**  **aOR (95%CI)** | **KS2 (*n=*6935)**  **aOR (95%CI)** | **KS4 (*n=*6935)**  **aOR (95%CI)** | **Across KS (*n=*20,805)**  **aOR (95%CI)** |
| --- | --- | --- | --- | --- | --- |
| Eye status | No eye condition | 1.00 | 1.00 | 1.00 | 1.00 |
|  | Strabismus alone | 0.74 (0.47-1.19) | 0.79 (0.51-1.25) | 0.87 (0.61-1.25) | 0.81 (0.64-1.03) |
|  | Refractive amblyopia | 1.23 (0.69-2.33) | 0.65 (0.39-1.11) | 0.78 (0.54-1.14) | 0.84 (0.64-1.10) |
|  | Strabismic/mixed amblyopia | 0.66 (0.26-1.93) | 0.59 (0.25-1.54) | 1.25 (0.63-2.55) | 0.89 (0.55-1.47) |
| Sex | Boys | 1.00 | 1.00 | 1.00 | 1.00 |
|  | Girls | 1.14 (0.94-1.38) | **0.77 (0.65-0.93)** | 1.11 (0.99-1.25) | 1.03 (0.94-1.12) |
| Ethnicity | Black/African/Caribbean | **0.54 (0.37-0.79)** | **1.56 (1.04-2.42)** | **1.62 (1.22-2.17)** | **1.22 (1.00-1.50)** |
|  | South Asian | **0.63 (0.49-0.81)** | 0.88 (0.69-1.14) | **2.04 (1.69-2.46)** | **1.23 (1.08-1.40)** |
|  | White | 1.00 | 1.00 | 1.00 | 1.00 |
|  | Other | 0.92 (0.62-1.40) | 1.25 (0.84-1.91) | **1.76 (1.34-2.34)** | **1.40 (1.15-1.72)** |
| Preterm birth | No | 1.00 | 1.00 | 1.00 | 1.00 |
|  | Yes | **0.68 (0.50-0.94)** | 0.93 (0.68-1.29) | 1.06 (0.84-1.34) | 0.92 (0.78-1.08) |
| Maternal education | A-levels or higher | 1.00 | 1.00 | 1.00 | 1.00 |
|  | O-levels | **0.57 (0.42-0.76)** | **0.71 (0.54-0.95)** | **0.58 (0.50-0.68)** | **0.60 (0.53-0.68)** |
|  | None | **0.33 (0.25-0.45)** | **0.44 (0.33-0.59)** | **0.37 (0.31-0.44)** | **0.37 (0.33-0.43)** |
| Household income quintile | 1 Richest | 1.00 | 1.00 | 1.00 | 1.00 |
|  | 2 | 1.05 (0.69-1.60) | 0.74 (0.50-1.10) | 1.07 (0.87-1.32) | 1.00 (0.84-1.18) |
|  | 3 | 0.76 (0.51-1.11) | **0.66 (0.44-0.96)** | 0.82 (0.67-1.01) | **0.78 (0.66-0.92)** |
|  | 4 | **0.66 (0.45-0.97)** | **0.57 (0.39-0.83)** | **0.60 (0.48-0.74)** | **0.61 (0.52-0.72)** |
|  | 5 Poorest | **0.58 (0.39-0.86)** | **0.47 (0.32-0.68)** | **0.39 (0.32-0.49)** | **0.46 (0.39-0.55)** |
| History of SEN at KS | No | 1.00 | 1.00 | 1.00 | 1.00 |
|  | Yes | **0.09 (0.07-0.11)** | **0.07 (0.06-0.09)** | **0.19 (0.17-0.22)** | **0.13 (0.12-0.14)** |
| Age | KS1 |  |  |  | 1.00 |
|  | KS2 |  |  |  | **1.22 (1.08-1.38)** |
|  | KS3 |  |  |  | **0.21 (0.19-0.24)** |

Odds ratios adjusted (aOR) for all covariates listed in the table and sample weights; *p*<0.05 in **bold**.
