# Supplementary material for: Educational attainment and trajectories at key stages of schooling for children with amblyopia compared to those without eye conditions: Findings from the Millennium Cohort Study
Source: PLoS One. 2023 Mar 30;18(3):e0283786. doi: 10.1371/journal.pone.0283786 (PMC10062655; doi:10.1371/journal.pone.0283786)
Supplement: S6 Table — (DOCX) [file pone.0283786.s007.docx]

**Table S6. Adjusted effects associated with passing GCSE (*n=*7422).**

| **Covariate** | **Category** | **aOR (95%CI)** |
| --- | --- | --- |
| Eye status | No eye condition | 1.00 |
|  | Strabismus alone | 1.07 (0.76-1.49) |
|  | Refractive amblyopia | 0.73 (0.51-1.06) |
|  | Strabismic/mixed amblyopia | 1.52 (0.79-2.97) |
| Sex | Boys | 1.00 |
|  | Girls | **1.37 (1.23-1.53)** |
| Ethnicity | Black/African/Caribbean | **1.36 (1.04-1.79)** |
|  | South Asian | **1.85 (1.56-2.20)** |
|  | White | 1.00 |
|  | Other | **1.35 (1.06-1.74)** |
| Preterm birth | No | 1.00 |
|  | Yes | 0.98 (0.79-1.21) |
| Maternal education | A-levels or higher | 1.00 |
|  | O-levels | **0.59 (0.52-0.68)** |
|  | None | **0.40 (0.34-0.46)** |
| Household income quintile | 1 Richest | 1.00 |
|  | 2 | 1.00 (0.84-1.19) |
|  | 3 | **0.75 (0.63-0.90)** |
|  | 4 | **0.54 (0.45-0.65)** |
|  | 5 Poorest | **0.41 (0.34-0.50)** |
| History of special education  needs at KS | No | 1.00 |
|  | Yes | **0.19 (0.17-0.22)** |

Odds ratios adjusted (aOR) for all covariates listed in the table and sample weights; *p*<0.05 in **bold**.
