# Supplementary material for: Educational attainment and trajectories at key stages of schooling for children with amblyopia compared to those without eye conditions: Findings from the Millennium Cohort Study
Source: PLoS One. 2023 Mar 30;18(3):e0283786. doi: 10.1371/journal.pone.0283786 (PMC10062655; doi:10.1371/journal.pone.0283786)
Supplement: S7 Table — (DOCX) [file pone.0283786.s008.docx]

**Table S7. Trajectories of adolescents’ university intentions.**

| Covariate | Category | Age 14 years (*n=*6581)  aOR (95%CI) | Age 17 years (*n=*6581)  aOR (95%CI) | Across ages (*n=*13,162)  aOR (95%CI) |
| --- | --- | --- | --- | --- |
| Eye status | No eye condition | 1.00 | 1.00 | 1.00 |
|  | Strabismus alone | 0.84 (0.54-1.30) | 1.14 (0.69-1.85) | 0.95 (0.68-1.31) |
|  | Refractive amblyopia | 1.06 (0.66-1.68) | 0.79 (0.42-1.42) | 0.93 (0.64-1.33) |
|  | Strabismic/mixed amblyopia | 1.14 (0.47-2.70) | 0.69 (0.22-1.95) | 0.92 (0.46-1.78) |
| Age | 14 years |  |  | 1.00 |
|  | 17 years |  |  | 0.95 (0.85-1.05) |
| Sex | Boys | 1.00 | 1.00 | 1.00 |
|  | Girls | **1.52 (1.33-1.74)** | **1.81 (1.56-2.11)** | **1.63 (1.48-1.81)** |
| Ethnicity | Black/African/Caribbean | **2.53 (2.05-3.12)** | **1.95 (1.53-2.50)** | **2.26 (1.77-2.91)** |
|  | South Asian | **2.75 (1.97-3.87)** | **1.78 (1.22-2.61)** | **2.22 (1.90-2.60)** |
|  | White | 1.00 | 1.00 | 1.00 |
|  | Other | **2.09 (1.57-2.81)** | 1.30 (0.93-1.80) | **1.69 (1.36-2.10)** |
| Preterm birth | No | 1.00 | 1.00 | 1.00 |
|  | Yes | 1.02 (0.77-1.34) | 0.93 (0.68-1.28) | 0.97 (0.79-1.19) |
| Maternal education | A-levels or higher | 1.00 | 1.00 | 1.00 |
|  | O-levels | **0.66 (0.56-0.77)** | **0.63 (0.53-0.76)** | **0.65 (0.58-0.73)** |
|  | None | **0.55 (0.45-0.67)** | 0.81 (0.64-1.01) | **0.65 (0.56-0.75)** |
| Household income quintile | 1 Richest | 1.00 | 1.00 | 1.00 |
|  | 2 | **0.72 (0.59-0.87)** | **0.77 (0.62-0.94)** | **0.74 (0.64-0.85)** |
|  | 3 | 0.85 (0.69-1.04) | 0.80 (0.64-1.01) | **0.83 (0.71-0.96)** |
|  | 4 | **0.61 (0.48-0.76)** | **0.65 (0.50-0.85)** | **0.63 (0.53-0.74)** |
|  | 5 Poorest | **0.66 (0.52-0.84)** | **0.66 (0.53-0.85)** | **0.66 (0.55-0.79)** |
| History of special education needs at Key Stage (KS) 2 and 4 | No | 1.00 | 1.00 | 1.00 |
|  | Yes | **0.73 (0.61-0.87)** | **0.60 (0.49-0.74)** | **0.68 (0.59-0.78)** |
| English at KS2 and KS4 | Not passed | 1.00 | 1.00 | 1.00 |
|  | Passed | 1.21 (0.86-1.71) | **1.66 (1.29-2.14)** | **1.49 (1.22-1.82)** |
| Mathematics at KS2 and KS4 | Not passed | 1.00 | 1.00 | 1.00 |
|  | Passed | **1.97 (1.46-2.66)** | **1.35 (1.05-1.74)** | **1.58 (1.30-1.91)** |
| Parents' university expectations  for child aged 14 and 17 years | Unlikely | 1.00 | 1.00 | 1.00 |
|  | Likely | **6.33 (5.05-8.00)** | **9.03 (7.42-11.06)** | **7.75 (6.68-9.02)** |

Odds ratios adjusted (aOR) for all covariates listed in the table and weighted for survey design; *p*<0.05 in **bold**.
