# Supplementary material for: Educational attainment and trajectories at key stages of schooling for children with amblyopia compared to those without eye conditions: Findings from the Millennium Cohort Study
Source: PLoS One. 2023 Mar 30;18(3):e0283786. doi: 10.1371/journal.pone.0283786 (PMC10062655; doi:10.1371/journal.pone.0283786)
Supplement: S8 Table — (DOCX) [file pone.0283786.s009.docx]

**Table S8. Complete case analysis of adolescents’ university intentions.**

| **Covariate** | **Category** | **Age 14 years (*n=*4279)**  **aOR (95%CI)** | **Age 17 years (*n=*4279)**  **aOR (95%CI)** | **Across ages (*n=*8558)**  **aOR (95%CI)** |
| --- | --- | --- | --- | --- |
| Eye status | No eye condition | 1.00 | 1.00 | 1.00 |
|  | Strabismus alone | 0.78 (0.46-1.30) | 1.08 (0.62-1.89) | 0.90 (0.62-1.31) |
|  | Refractive amblyopia | 1.20 (0.61-2.35) | 0.88 (0.42-1.84) | 1.01 (0.62-1.65) |
|  | Strabismic/mixed amblyopia | 1.18 (0.53-2.85) | 0.60 (0.16-2.09) | 1.00 (0.44-2.26) |
| Sex | Boys | 1.00 | 1.00 | 1.00 |
|  | Girls | **1.44 (1.22-1.70)** | **1.59 (1.33-1.91)** | **1.51 (1.34-1.7)** |
| Ethnicity | Black/African/Caribbean | **2.47 (1.88-3.26)** | **2.84 (2.08-3.89)** | **2.59 (2.11-3.18)** |
|  | South Asian | **3.51 (2.22-5.74)** | **2.32 (1.42-3.88)** | **2.95 (2.11-4.19)** |
|  | White | 1.00 | 1.00 | 1.00 |
|  | Other | **2.13 (1.48-3.10)** | **1.57 (1.05-2.37)** | **1.85 (1.41-2.43)** |
| Preterm birth | No | 1.00 | 1.00 | 1.00 |
|  | Yes | 0.90 (0.64-1.27) | 1.10 (0.75-1.61) | 0.97 (0.75-1.26) |
| Maternal education | A-levels or higher | 1.00 | 1.00 | 1.00 |
|  | O-levels | **0.64 (0.53-0.78)** | **0.58 (0.47-0.71)** | **0.61 (0.53-0.71)** |
|  | None | **0.50 (0.39-0.65)** | 0.84 (0.64-1.10) | **0.63 (0.53-0.76)** |
| Household income quintile | 1 Richest | 1.00 | 1.00 | 1.00 |
|  | 2 | **0.69 (0.54-0.86)** | **0.67 (0.52-0.85)** | **0.68 (0.57-0.80)** |
|  | 3 | 0.87 (0.67-1.12) | **0.69 (0.53-0.90)** | **0.78 (0.65-0.93)** |
|  | 4 | **0.63 (0.47-0.84)** | **0.58 (0.43-0.80)** | **0.61 (0.49-0.75)** |
|  | 5 Poorest | **0.67 (0.49-0.91)** | **0.71 (0.50-1.00)** | **0.68 (0.54-0.86)** |
| History of special education needs at Key Stages (KS) 2 and 4 | No | 1.00 | 1.00 | 1.00 |
|  | Yes | 0.83 (0.65-1.06) | **0.54 (0.42-0.68)** | **0.68 (0.57-0.80)** |
| English at KS2 and KS4 | Not passed | 1.00 | 1.00 | 1.00 |
|  | Passed | 0.75 (0.47-1.20) | **1.51 (1.13-2.02)** | 1.23 (0.96-1.57) |
| Mathematics at KS2 and KS4 | Not passed | 1.00 | 1.00 | 1.00 |
|  | Passed | **2.27 (1.55-3.35)** | 1.30 (0.97-1.75) | **1.59 (1.27-2.01)** |
| Parents' university expectation  for child aged 14 and 17 years | Unlikely | 1.00 | 1.00 | 1.00 |
|  | Likely | **9.12 (6.69-12.70)** | **8.79 (7.08-10.98)** | **8.49 (7.12-10.17)** |
| Age | 14 years |  |  | 1.00 |
|  | 17 years |  |  | **1.56 (1.38-1.77)** |

Odds ratios adjusted (aOR) for all covariates listed in the table and sample weights; *p*<0.05 in **bold**.
